# Characterization of the bile and gall bladder microbiota of healthy pigs

**DOI:** 10.1002/mbo3.218

**Published:** 2014-10-21

**Authors:** Esther Jiménez, Borja Sánchez, Annarita Farina, Abelardo Margolles, Juan M Rodríguez

**Affiliations:** 1 Departamento de Nutrición, Bromatología y Tecnología de los Alimentos, Universidad Complutense de Madrid28040, Madrid, Spain; 2 Department of Analytical Chemistry and Food Science, Faculty of Food Science and Technology, University of Vigo – Ourense Campus32004, Ourense, Spain; 3 Department of Human Protein Sciences, University of GenevaCH-1211, Geneva, Switzerland; 4 Instituto de Productos Lácteos de Asturias (IPLA-CSIC)Paseo Río Linares s/n, 33300, Villaviciosa, Spain

**Keywords:** Bile, gall bladder, microbiome, microbiota, proteome

## Abstract

Bile is a biological fluid synthesized in the liver, stored and concentrated in the gall bladder (interdigestive), and released into the duodenum after food intake. The microbial populations of different parts of mammal's gastrointestinal tract (stomach, small and large intestine) have been extensively studied; however, the characterization of bile microbiota had not been tackled until now. We have studied, by culture-dependent techniques and a 16S rRNA gene-based analysis, the microbiota present in the bile, gall bladder mucus, and biopsies of healthy sows. Also, we have identified the most abundant bacterial proteins in the bile samples. Our data show that the gall bladder ecosystem is mainly populated by members of the phyla *Proteobacteria*, *Firmicutes*, and *Bacteroidetes*. Furthermore, fluorescent in situ hybridization (FISH) and transmission electron microscopy (TEM) allowed us to visualize the presence of individual bacteria of different morphological types, in close association with either the epithelium or the erythrocytes, or inside the epithelial cells. Our work has generated new knowledge of bile microbial profiles and functions and might provide the basis for future studies on the relationship between bile microbiota, gut microbiota, and health.

## Introduction

The gastrointestinal tract (GIT) of humans and other mammals harbors a complex microbiota that confers immunological, metabolic, and neurological benefits to the host (Bäckhed et al. 2005; Gill et al. 2006; Xu et al. 2007; Collins et al. 2012). Within the GIT, most of the studies regarding bacterial diversity and functions have been focused on the colon because of the easy access to samples (either feces or biopsies) and also, the very high bacterial abundance in such location (˜10^12^ bacteria per gram of colon content). In contrast, the characterization of the microbiota of other GIT locations (stomach, small intestine) has been hampered by technical and ethical difficulties to access biological samples in healthy hosts. However, culture-independent methods have revealed an unexpectedly wide microbial diversity in such locations (Bik et al. 2006; Stearns et al. 2011; Zoetendal et al. 2012; Wang and Yang 2013), and have suggested an important role of such site-specific microbiomes in the host physiology (Li et al. 2009; Yang et al. 2013).

Traditionally, the stomach was considered as a particularly hard barrier for orally ingested microorganisms and, as a consequence, was long thought to be a sterile environment. However, the discovery of *Helicobacter pylori* led to a paradigm shift in our understanding of the stomach as an ecological niche for bacteria (Fox and Sheh 2013). Nowadays, a substantial body of evidence shows that some bacterial phyla (including *Firmicutes*, *Actinobacteria*, *Bacteroidetes*, and *Proteobacteria*) and genera (*Lactobacillus*, *Streptococcus*, *Propionibacterium*, etc.) can be regularly isolated from the stomachs of healthy adults (Yang et al. 2013).

Similar to the stomach, the gall bladder (one of the accessory digestive glands) has also been considered a very hostile territory for bacteria. Bile, whose major constituents include bile acids, cholesterol, phospholipids, and the pigment biliverdin, functions as a biological detergent that emulsifies and solubilizes lipids, being essential for fat digestion. This detergent property of bile also confers potent antimicrobial activity, primarily through the dissolution of bacterial membranes (Begley et al. 2005a, 2005b). In addition, the antimicrobial activity of some of its compounds, such as bile acids, seems to play a key role to control bacterial proliferation in the small intestine (Hofmann and Eckmann 2006; Inagaki et al. 2006). In fact, those conditions that are linked to a decrease in bile acid secretion are usually associated with bacterial overgrowth in the small intestine and, eventually, with infection (Bauer et al. 2001).

Despite bacterial hostility of bile, it has been recently shown that strains of some species, such as *Salmonella* spp. or *Listeria monocytogenes*, are able to grow and survive within the gall bladder and may be associated with infections and with the pathogenesis of gallstones (Hardy et al. 2004, 2006; Crawford et al. 2008, 2010; Dowd et al. 2011; Gonzalez-Escobedo et al. 2011). These studies showed that, at least, some bacteria can colonize and survive in the gall bladder. However, the global bacterial diversity of bile and gall bladder from healthy hosts has not been assessed yet.

In this context, the objectives of this work were to describe the microbiota (culture-dependent methods), the microbiome (culture-independent methods), and the microbial protein content in bile and gall bladder samples collected from healthy pigs.

## Experimental Procedures

### Sample collection

Six gall bladders were taken from six healthy white sows (same age and farm) that were sacrificed in a conventional slaughterhouse (Matadero Madrid Norte, San Agustín de Guadalix, Madrid) under the supervision of the official Veterinary personnel. Previously, the procedure was approved by the Ethical Committee on Animal Experimentation of the Complutense University of Madrid (Spain). Once the abdominal cavities of the animals were open, the gall bladders were clumped, removed from the carcasses using sterile scalpels, soaked in a clorhexidine solution, introduced in independent sterile containers (which were kept on ice), and transported to the laboratory within the first hour. Then, each bladder was rinsed with sterile PBS (pH 7.2) and bile (˜50 mL) was extracted using a 20-mL sterile syringe after the application of hydrogen peroxide in the zone where the needle was inserted. Once the bladder was completely emptied, the superficial mucus layer coating the gall bladder epithelium was collected and then, three biopsies of 1 cm^2^ were cut with a scalpel. Swabs from the external side of each gall bladder were obtained, before they were clumped in order to confirm that there was no artificial contamination due to the abattoir environment.

### Culture-dependent assessment of bacterial diversity

The swabs from the external side of the gall bladders, the bile of one sow, and the gall bladder mucus and biopsies of the remaining five sows were cultured. The bile of the later animals was reserved for metagenomic and proteomic analyses. Biopsy specimens were cut into small pieces and homogenized with 10 mL of sterile PBS (pH 7.2). The samples were plated onto Brain Heart Infusion for total counts (BHI; Oxoid, Basingstoke, UK), Columbia Blood Agar for isolation of streptococci, staphylococci, enterococci, corynebacteria, and related Gram-positive bacteria (CNA; BioMerieux, Marcy l'Etoile, France), Man, Rogosa, Sharpe for isolation of lactic acid bacteria (MRS; Oxoid) and McConkey (MCK, BioMerieux; for enterobacteria) agar plates and incubated aerobically at 37°C for 48 h. In parallel, samples were also plated onto MRS (Oxoid) agar plates supplemented with l-cysteine (0.5 g/L) (MRS-cys), which were incubated anaerobically (85% nitrogen, 10% hydrogen, 5% carbon dioxide) in an anaerobic workstation (MINI-MACS; DW Scientific, Shipley, UK) at 37°C for 48 h.

### Identification of the isolates

All isolates were observed by optical microscopy to determine their morphology and Gram staining. Additionally, they were tested for catalase, oxidase, and coagulase activities. Finally, they were identified by MALDI-TOF (VITEK-2) in the facilities of ProbiSearch (Tres Cantos, Spain) or by PCR sequencing of a 470 bp fragment of the 16S rRNA gene using primers pbl16 (5′-AGAGTTTGATCCTGGCTCAG-3′) and mbl16 (5′-GGCTGCTGGCACGTAGTTAG-3′). The amplicons were purified using the Nucleospin Extract II kit (Macherey–Nagel, Duren, Germany) and sequenced at the Genomics Unit of the Scientific Park of Madrid, Spain.

### Fluorescent in situ hybridization analysis

The universal eubacterial oligonucleotide probe EUB-338 (5′-GCT GCC TCC CGT AGG AGT-3′), labeled at the 5′ end with fluorochrome 6-FAM (carboxyfluorescein), and the irrelevant control probe nonEUB-338 (5′-ACT CCT ACG GGA GGC AGC-3′), complementary to EUB-338 labeled with the fluorochrome Cy5, both synthesized by Eurofins MWG (Ebersberg, Germany), were used for in situ hybridization with bacterial DNA on a glass surface.

For each hybridization reaction, the biopsy specimen was shock frozen in Tissue-Tek freezing medium (Sakura Finetek Europe, Zoeterwoude, The Netherlands), cut into 10-*μ*m thick sections using a cryostat (Leica, Wetzlar, Germany), and placed on Superfrost Plus glass slides (Menzel GmbH & Co KG, Braunschweig, Germany). The biopsies were fixed with fresh and cold 4% (v/v) paraformaldehyde (Electron Microscopy Science, Hatfield, USA) solution in PBS pH 7.2 for 1 h at 4°C and then, washed with PBS to remove residual fixative solution. After air drying of the specimens, three tissue sections of each biopsy were overlaid with 100 *μ*L of permeabilization buffer (100 mmol/L Tris-HCl [Sigma-Aldrich, St Louis, MO], 50 mmol/L Ethylenediaminetetraacetic acid (EDTA) [Sigma] pH 8.0, lysozyme 10 mg/mL [Sigma], proteinase K 100 *μ*g/mL [Sigma], and lysostaphin 10 *μ*g/mL [Sigma]) during 45 min at 37°C. Enzymatic treatments were stopped by rinsing the slides thoroughly with sterile water and drying. Hybridization buffer (0.9 mol/L NaCl, 100 mmol/L Tris-HCl [pH 7.6], 0.1% sodium dodecyl sulfate) containing 35% formamide (Sigma) and 5 ng/*μ*L of the oligonucleotide probe EUB-338 or nonEUB-338 (labeled at the 5′ end with 6-carboxyfluorescein and rhodamine 6G, respectively) was spotted onto the section. The slides were incubated in a dark, humid chamber at 50°C overnight and, after that, were washed at the same temperature in a prewarmed buffer containing 0.9 mol/L NaCl, 20 mmol/L Tris-HCl (pH 8.0) for 30 min. After that time, the slides were briefly washed with sterile water, air-dried, and stored in the dark.

Subsequently, samples were blue stained with DAPI (49,69-diamidino-2-phenylindole; Sigma), which detects the DNA of bacteria, fungi, and host cells. ProLong Gold antifade reagent (Invitrogen, Ltd., Paisley, UK) was used as a mounting medium on hybridized slides. Finally, the slides were analyzed by confocal scanning laser microscopy (CSLM) using a TCS SP-2 microscope (Leica Lasertechnik, Heidelberg, Germany). Confocal images were obtained using the 63× (numeric aperture 1.4) oil immersion objective. Composite images were produced combining two filters so that the nuclei of the gall bladder epithelial cells appeared blue (DAPI staining) while bacteria appeared green due to the hybridization with the EUB338 probe.

### Transmission electron microscopy analysis

Biopsies were fixed in 4% (v/v) paraformaldehyde and 2.5% (v/v) glutaraldehyde in phosphate buffered saline (PBS) pH 7.2 for 4 h at 4°C. The samples were washed with cold sterile PBS every 20 min, at least four times. Biopsies were then postfixed with 1% (w/v) EM grade osmium tetroxide solution for 90 min at room temperature. This was followed by washing with sterile deionized water four times every 15 min and a complete dehydration of the specimens in a series of increasing acetone concentrations (30%, 50%, 70%, 80%, 90%, 95%, and 100%). The samples were then infiltrated and embedded gradually in epoxy resin. Ultrathin sections were obtained in an Ultracut E microtome (Reichert Jung) and stained with 2% uranyl acetate followed by Reynold's lead citrate. Electron micrographs were obtained with a JEOL 1010 electron microscope at 100 kV and equipped with a CCD megaview camera at the Centro Nacional de Microscopía (Complutense University of Madrid, Spain).

### DNA extraction and 16S rRNA metagenomic data generation

DNA was extracted from bile samples (1 mL) of four animals and also, from gall bladder mucus samples of two of these animals, following a protocol described previously (Moles et al., 2013). The DNA yield was measured using a NanoDrop® ND-1000 UV spectrophotometer (Nano-Drop Technologies, Wilmington, DE).

The DNA samples were used for a 16S rRNA sequence-based survey of bacterial diversity. Amplicons of ˜345 bp from the V1 to V2 hypervariable region of 16S rRNA genes were generated by PCR using 27F-DegL (5′-GTTYGATYMTGGCTCAG-3′) in combination with an equimolar mixture of two reverse primers, 338R-I (5′-GCWGCCTCCCGTAGGAGT-3′) and 338R-II (5′GCWGCCACCCGTAGGTGT-3′) for each DNA extraction.

PCR amplification, pool emulsion, and 454 pyrosequencing were performed at the Genomics Unit of the Fundación Parque Científico de Madrid (Spain) on a 454 Life Sciences Genome Sequencer FLX-454-Titanium machine (Roche, Basel, Switzerland).

The analysis and taxonomic assignment of the 16S rDNA metagenomic data was done using the MG7 program developed by Era7 Bioinformatics (https://github.com/pablopareja/MG7), which uses cloud computing for the parallel massive BLAST similarity analysis to infer both, function and taxonomic assignment. MG7 taxonomic assignment was done based on two different paradigms: Best Blast Hit (BBH) and Lowest Common Ancestor (LCA). In the first case, the assignment of each read was made to the organism to which corresponds the BBH obtained after searching the *nt* database (NCBI), while LCA did it to the lowest node in the taxonomic tree that is ancestor of the top 10 BBHs. Once the similarity data were obtained, the results were organized in the graph database. Considering the presence of large metagenomic data sets with nonspecific taxonomic assignment in the *nt* database, assignment based on BBH was considered to be more specific and informative than that based on LCA.

The data set obtained in this work is available in the European Nucleotide Archive repository, under the study accession number: PRJEB6268 (http://www.ebi.ac.uk/ena/data/view/PRJEB6268).

The bacterial richness and diversity of the samples were determined by calculating the Shannon–Weaver diversity index, which takes into account the number and evenness of the bacterial species.

### Functional inference analysis

The functionality of the different bile metagenomes was predicted using the software PICRUSt 1.0.0 (http://picrust.github.com) (Langille et al. 2013). In short, this software allows the prediction of functional KEGG pathway abundances from the 16S rDNA-based metagenomes. First, a collection of closed reference operational taxonomic units (OTU) was obtained from the filtered reads using QIIME v1.7.0 (Caporaso et al. 2010) by querying the data against the GreenGenes database (version 13.5, May 2013, http://greengenes.secondgenome.com). Reverse strand matching was enabled during the query and OTUs were picked at a 97% identity. A BIOM-formatted table (Biological Observation Matrix, McDonald et al. 2012) was obtained with the pick_closed_reference_otus.py script. This table, containing the relative abundances of the different reference OTUs in all the metagenomes, was normalized using the predicted 16S rDNA copy number with the script normalize_by_copy_number.py. Final functional predictions, inferred from the metagenomes, were created with the script predict_metagenomes.py. Predicted metagenomic contents were collapsed at the three hierarchical KEGG pathway levels (http://www.genome.jp/kegg/pathway.html) with the categorize_by_function.py script and tables were exported in tab-delimited text format for further analysis.

### Protein extraction and precipitation

Five hundred *μ*L of bile, obtained as mentioned before, was diluted with seven volumes of sterile PBS and centrifuged at 200,000*g* for 30 min at 4°C. Pellets, containing debris and microorganisms, were resuspended in 500 *μ*L of PBS and proteins were extracted with a combination of zirconia beads/sonication treatment. First, resuspended bile pellets were submitted to a disruption step by adding 0.1 mm zirconium–silica beads (Biospec Products, Bartlesville, OK), followed by three 1 min pulses at maximum speed in a bead beater (FastPrep FP120 Thermo Savant; Qbiogene, Inc., Illkirch, France) with two intervals of 1 min in which samples were cooled on ice. Second, samples were sonicated in ice using a CV17 sonicator device (VibraCell, Sonics & Materials Inc., Danbury, CT) by three 1 min pulses with two periods of 1 min in which samples were allowed to cool down in ice.

Samples were centrifuged at 16,000*g* for 10 min at 4°C, and proteins were precipitated from the cell-free supernatants using a standard trichloroacetic acid (TCA)/acetone protocol. Briefly, samples were mixed with 10 volumes of cold 10% TCA in acetone (previously stored at –20°C), vortexed, and incubated overnight at −20°C. Samples were centrifuged at 16,000*g* for 10 min at 4°C and one volume of cold acetone was added to pellets. The mixture was vortexed again, incubated for 10 min at −20°C, and centrifuged at 16,000*g* for 10 min at 4°C. Finally, the supernatants were removed and pellets were allowed to air dry.

### In solution tryptic digestion

Samples, containing around 1 mg of total proteins, were resuspended in 500 *μ*L of 50 mmol/L NH_4_HCO_3_. Proteins were reduced by incubating with the presence of dithiothreitol (DTT) 5.8 mmol/L at 95°C for 5 min and thus alkylated by incubating with iodoacetamide (IAA) 11.7 mmol/L 15 min at RT in the dark. Trypsin (Promega Biotech Ibérica S.L., Alcobendas Spain) 10 ng/mL was added in each sample and incubation progressed on at 37°C. Finally, samples were centrifuge at 16,000*g* for 10 min and dried in a vacuum centrifuge (Concentrator 5301, Eppendorf AG, Hamburg, Germany).

### Mass spectrometry analysis

Electrospray ionization (ESI) linear trap quadrupole (LTQ)-orbitrap (OT) mass spectrometry (MS) was performed on a LTQ Orbitrap Velos Pro from Thermo Electron (San Jose, CA) equipped with a NanoAcquity system from Waters (Waters Corporation, Manchester, UK). Peptides were trapped on a homemade 5 *μ*m 200 Å Magic C18 AQ (Michrom, Auburn, CA) 0.1 × 20 mm precolumn and separated on a homemade 5 *μ*m 100 Å Magic C18 AQ (Michrom) 0.75 × 150 mm column with a gravity-pulled emitter. The analytical separation was run for 65 min using a gradient of H_2_O/FA 99.9%/0.1% (solvent A) and CH_3_CN/FA 99.9%/0.1% (solvent B). The gradient was run at a flow rate of 220 nL/min as follows: 5% B for 1 min, from 5% to 35% B in 54 min, from 35% to 80% B in 10 min. For MS survey scans, the Orbitrap resolution was set to 60,000 and the ion population was set to 5 × 10^5^ with an *m/z* window from 400 to 2000. Eight precursor ions were selected for collision-induced dissociation (CID) in the LTQ. For this, the ion population was set to 7 × 10^3^ (isolation width of 2 *m/z*). The normalized collision energies were set to 35% for CID.

### Protein database searching

Peak lists were generated from raw data by using EasyProtConv (Gluck et al. 2013). All MS/MS samples were analyzed using Mascot (version 2.2.0; Matrix Science, London, UK). Mascot was setup to search the UniProt release 2014_01 database selected for Bacteria and *Sus scrofa* (40052542 entries) assuming the digestion enzyme trypsin (max missed cleavages: 1). Mascot was searched with a fragment ion mass tolerance of 0.60 Da and a parent ion tolerance of 10 PPM. Carbamidomethylation of cysteine was specified in Mascot as a fixed modification. Oxidation of methionine was specified in Mascot as a variable modification.

### Criteria for protein identification and taxonomic analysis

Scaffold (version 4.3.0, Proteome Software Inc., Portland, OR) was used to validate MS/MS-based peptide and protein identifications. Peptide identifications were accepted if they could be established at greater than 90% probability by the Peptide Prophet algorithm (Keller et al. 2002) with Scaffold delta-mass correction. Protein identifications were accepted if they could be established at greater than 90% probability and contained at least 1 identified peptide in two or more injections. Protein probabilities were assigned by the Protein Prophet algorithm (Nesvizhskii et al. 2003). Proteins sharing significant peptide evidence were grouped into clusters. Proteins that contained similar peptides and could not be differentiated based on MS/MS analysis alone were grouped to satisfy the principles of parsimony. The taxonomic analysis of bacterial entries univocally attributable to a definite phylum was performed by using the retrieve module of the UniProt Protein knowledgebase (http://www.uniprot.org/).

## Results

### Detection of live bacteria in bile, gall bladder mucus, and biopsies

No bacterial growth was detected in the swabs obtained from the external surface of the gall bladders, indicating that there was no artificial contamination due to the abattoir environment. In contrast, inoculation of the bile sample led to bacterial growth in all the culture media tested. In the case of the gall bladder mucus layer and biopsies from the other five animals, all the samples led to bacterial growth on almost all the culture media tested, with the exception of the anaerobic cultured media MRSc where growth was scarce (Table 1). Bacterial counts in the different samples and animals oscillated between 2.7 and 4.8 log_10_ cfu/g (Table 1). *Streptococcus alactolyticus* was the dominant species (˜40% of the isolates) in bile of animal 6, followed by *Staphylococcus epidermidis* and *Corynebacterium testudinoris*. In addition seven other species were isolated from the bile sample: *Enterococcus faecalis*, *Kocuria rhizophila*, *Macrococcus caseolyticus*, *Bacillus sporothermodurans*, *Lactobacillus amylovorus*, *Lactobacillus reuteri*, and *Escherichia coli*.

**Table 1:** Bacterial counts (log_10_ cfu/g) obtained after culturing porcine bile, gall bladder mucus (M), and gall bladder biopsies (B) on different agar media

Animal	Sample	BHI	CNA	MCK	MRS	MRSc
1	M	2.70	3.30	2.70		
	B	3.54	3.30	2.70	–	–
2	M	3.30	3.40	3.00	–	–
	B	3.18	3.00	–	–	–
3	M	3.98	4.15	3.85	–	–
	B	3.90	3.65	3.48	3.18	–
4	M	4.60	4.23	–	4.78	4.56
	B	4.51	4.57	–	4.54	4.81
5	M	2.70	3.00	–	–	–
	B	–	2.70	–	2.70	–
6	Bile	3.98	4.05	2.85	3.18	3.08

M, gall bladder mucus layer; B, gall bladder biopsy; BHI, Brain Heart Infusion; CNA, Columbia Blood Agar; MCK, McConkey; MRS, Man, Rogosa, Sharpe; MRSc, Man, Rogosa, Sharpe supplemented with l-cysteine.

The species that were identified in the gall bladder mucus layer and biopsies of animals 1–5 are shown in Table 2. They belonged to the phyla *Actinobacteria* (32%), *Proteobacteria* (32%), *Firmicutes* (34%), and *Bacteroidetes* (2%). At the genus level, *Kocuria* and *Rothia* (phylum *Actinobacteria*), *Acinetobacter* and *Psycrhobacter* (phylum *Proteobacteria*), and *Staphylococcus* and *Streptococcus* (phylum *Firmicutes*) could be detected in three different samples. Globally, the number of species that grew in the agar media included in this study oscillated from 3 to 20 per sample (samples 5 and 3, respectively).

**Table 2:** Species isolated from gall bladder mucus and biopsies in this study

Sample	Phylum	Class	Species	Location
1	*Actinobacteria*	*Actinobacteria*	*Corynebacterium auriscanis*	M
	*Actinobacteria*	*Actinobacteria*	*Corynebacterium suicordis*	B
	*Actinobacteria*	*Actinobacteria*	*Corynebacterium testudinoris*	B
	*Actinobacteria*	*Actinobacteria*	*Kocuria rhizophila*	B
	*Actinobacteria*	*Actinobacteria*	*Rothia mucilaginosa*	B
	*Proteobacteria*	*Alphaproteobacteria*	*Brevundimonas* sp.	B
	*Proteobacteria*	*Alphaproteobacteria*	*Paracoccus alcaliphilus*	M
	*Proteobacteria*	*Gammaproteobacteria*	*Psychrobacter sanguinis*	B
2	*Actinobacteria*	*Actinobacteria*	*Kocuria rhizophila*	M
	*Actinobacteria*	*Actinobacteria*	*Rothia mucilaginosa*	M, B
	*Firmicutes*	*Bacilli*	*Exiguobacterium acetylicum*	M
	*Firmicutes*	*Bacilli*	*Staphylococcus aureus*	M
	*Firmicutes*	*Bacilli*	*Staphylococcus warneri*	B
	*Firmicutes*	*Bacilli*	*Streptococcus pluranimalium*	B
	*Proteobacteria*	*Gammaproteobacteria*	*Acinetobacter lwoffii*	M
	*Proteobacteria*	*Gammaproteobacteria*	*Moraxella osloensis*	M
	*Proteobacteria*	*Gammaproteobacteria*	*Psychrobacter sanguinis*	M
3	*Actinobacteria*	*Actinobacteria*	*Corynebacterium suicordis*	B
	*Actinobacteria*	*Actinobacteria*	*Kocuria rhizophila*	M
	*Actinobacteria*	*Actinobacteria*	*Leucobacter alluvii*	M
	*Actinobacteria*	*Actinobacteria*	*Microbacterium oxydans*	M, B
	*Actinobacteria*	*Actinobacteria*	*Microbacterium* sp.	B
	*Actinobacteria*	*Actinobacteria*	*Rothia* sp.	M, B
	*Bacteroidetes*	*Bacteroidetes*	*Wautersiella falsenii*	M
	*Firmicutes*	*Bacilli*	*Exiguobacterium acetylicum*	M
	*Firmicutes*	*Bacilli*	*Exiguobacterium indicum*	B
	*Firmicutes*	*Bacilli*	*Lactococcus garvieae*	B
	*Firmicutes*	*Bacilli*	*Macrococcus caseolyticus*	B
	*Firmicutes*	*Bacilli*	*Staphylococcus epidermidis*	B
	*Firmicutes*	*Bacilli*	*Streptococcus alactolyticus*	M, B
	*Proteobacteria*	*Gammaproteobacteria*	*Acinetobacter johnsonii*	M
	*Proteobacteria*	*Gammaproteobacteria*	*Acinetobacter lwoffii*	B
	*Proteobacteria*	*Gammaproteobacteria*	*Acinetobacter* sp.	M
	*Proteobacteria*	*Gammaproteobacteria*	*Chryseobacterium* sp.	B
	*Proteobacteria*	*Gammaproteobacteria*	*Enterobacter amnigenus*	M, B
	*Proteobacteria*	*Gammaproteobacteria*	*Pantoea* sp.	M, B
	*Proteobacteria*	*Gammaproteobacteria*	*Psychrobacter sanguinis*	M, B
4	*Actinobacteria*	*Actinobacteria*	*Microbacterium* sp.	M
	*Firmicutes*	*Bacilli*	*Lactococcus garvieae*	M, B
	*Firmicutes*	*Bacilli*	*Streptococcus alactolyticus*	M, B
	*Proteobacteria*	*Gammaproteobacteria*	*Acinetobacter* sp.	B
5	*Firmicutes*	*Bacilli*	*Staphylococcus pasteuri*	B
	*Firmicutes*	*Bacilli*	*Streptococcus alactolyticus*	M
	*Firmicutes*	*Bacilli*	*Streptococcus dysgalactiae*	M

M, gall bladder mucus layer; B, gall bladder biopsy.

### Fluorescent in situ hybridization and transmission electron microscopy analysis

**Figure 1:**
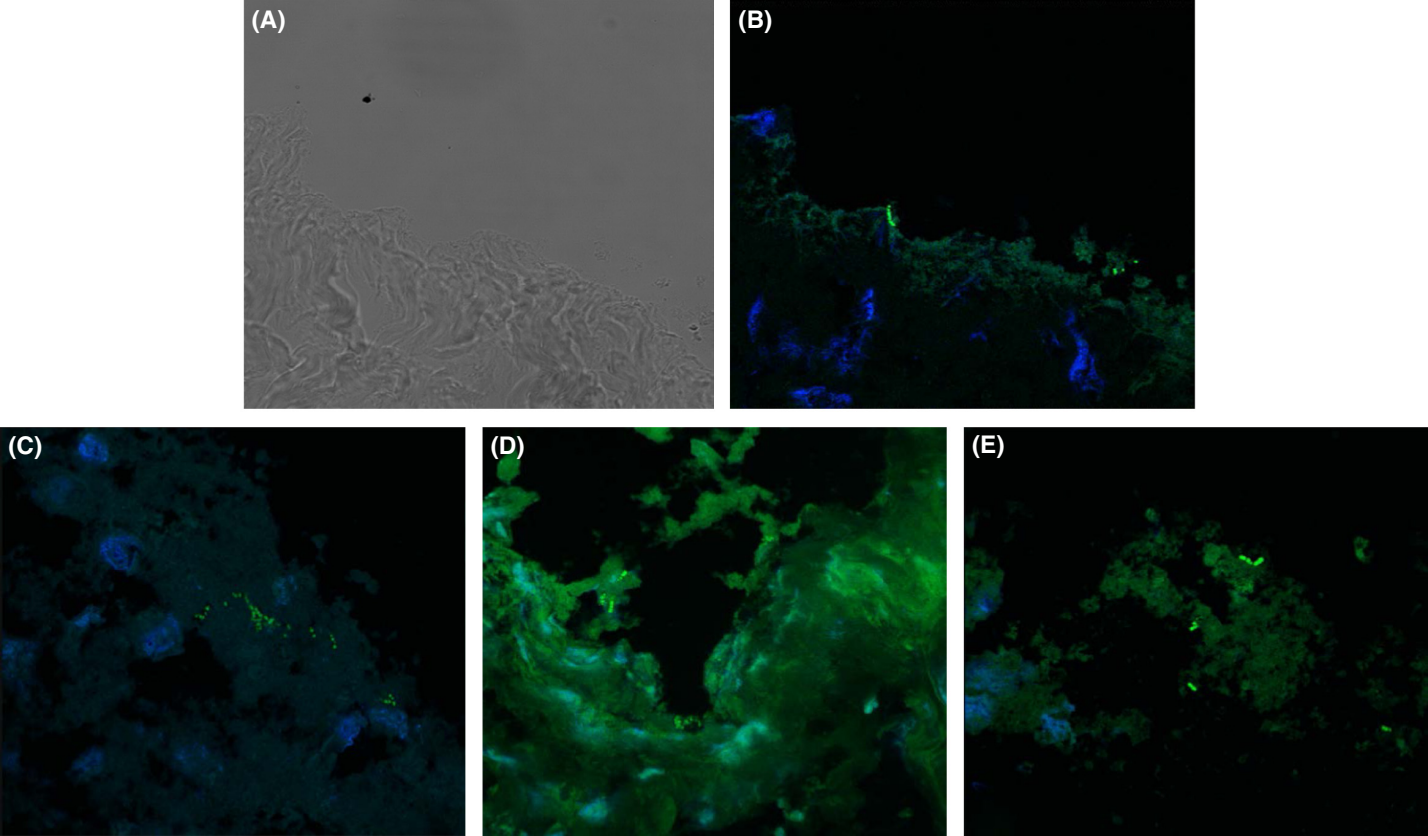
FISH in situ localization of bacteria in gall bladder biopsies. The nuclei of the gall bladder epithelial cells appeared blue due to DAPI staining while bacteria appeared green due to the hybridization with the EUB338 probe. Figure shows the same section observed by using (A) bright field microscopy and (B) a composition of two filters, one for DAPI blue detection and the other for detection of green FAM fluorescence. The figure suggests the presence of bacteria inside the host cells (Fig. 1C and D) and in the borders of the section (Fig. 1E).

**Figure 2:**
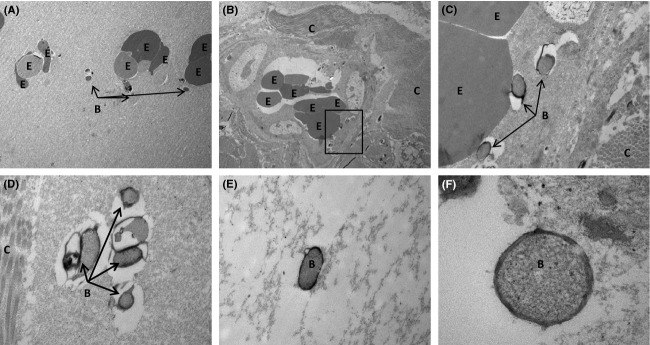
TEM ultramicrophotographic images of bacteria within the gall bladder tissue. A and B show bacteria-like structures closely associated to erythrocytes, while C–F show potential gall bladder bacteria at a higher magnification. Magnification: (A) 6000×, (B) 5000×, (C) 25,000×, (D and E) 40,000×, and (F) 150,000×. B, bacteria-like structures; E, erythrocyte; C, collagen fibers.

The presence of individual bacteria was visualized with the green FAM fluorescence in the gall bladder biopsy samples obtained from the sows (Fig. 1). Different morphological types of bacteria were observed (Fig. 1) and most of them were located in the border of the tissue (Fig. 1A, B, and E) although a few seemed to be inside the epithelial cells, as indicated by their close associated to the host cell nuclei (Fig. 1C and D). Similarly to the fluorescent in situ hybridization (FISH) analysis, some structures that were compatible with bacteria could be observed in the gall bladder biopsies by transmission electron microscopy (TEM) (Fig. 2). Interestingly, by using this technique, some bacterial-like structures could be seen in close association with erythrocytes within the gall bladder tissue (Fig. 2A–C).

### Bile and gall bladder mucus microbiomes

**Figure 3:**
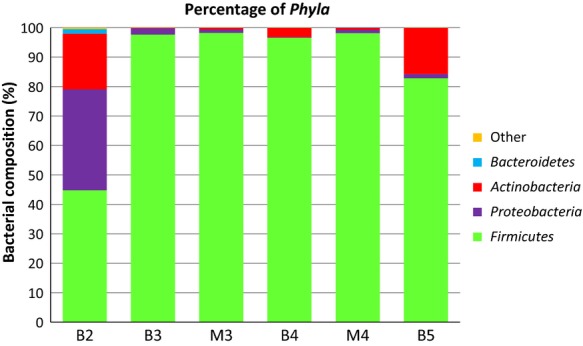
Best hit comparison of bacterial phyla in the four bile (B) and two mucus (M) samples analyzed by pyrosequencing in this study. The percent of sequences of each phyla was assigned according to the Best Blast Hit paradigm.

**Figure 4:**
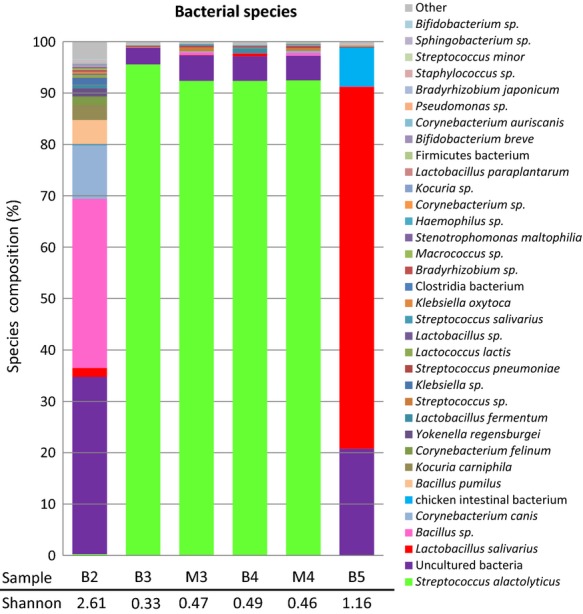
Species for which DNA was detected in the four bile (B) and two mucus (M) samples analyzed by pyrosequencing in this study. Bacterial diversity was assessed using the Shannon–Weaver diversity index.

The DNA extracted from the bile of four animals (2–5) and that from the gall bladder mucus layer of two of them (3 and 4) were analyzed by pyrosequencing. The V1–V2 region of the bacterial 16S rRNA gene was amplified from the DNA using universal primers, and barcoded pyrosequencing of the amplicons produced more than 50 Mbp. An average of ˜48,000 sequences (length: 324 ± 55 bp) were acquired for the bile samples, while 7160 and 4776 sequences were obtained from the mucus samples of animals 3 and 4, respectively. *Firmicutes* was the main phylum detected in all the samples (Fig. 3). More than 90% of the reads obtained in samples 3 and 4 corresponded to *Str. alactolyticus* DNA while that of *Lactobacillus salivarius* dominated in sample 5 (Fig. 4). Gall bladder 2 showed the highest diversity (212 different species detected) followed by gall bladder 5 (Fig. 4). The number of species that represented >0.1% of the reads oscillated between 17 and 34 (bile from animals 3 and 2, respectively) (Table 3). In relation to the bacterial diversity, no differences were observed when the reads obtained in bile and mucus samples of animals 3 and 4 were compared using the Shannon–Weaver diversity index (Fig. 4).

**Table 3:** Bacterial species which DNA was detected in the bile and gall bladder mucus samples

Species	Sample					
	B 2	B 3	M3	B 4	M4	B 5
*Bacillus pumilus*	4.81	–	0.03	–	0.02	–
*Bacillus* sp.	32.88	0.01	0.47	–	0.51	0.01
*Bifidobacterium breve*	0.22	–	–	–	–	–
*Bifidobacterium* sp.	0.11	–	–	0.01	0.02	–
*Bradyrhizobium japonicum*	0.16	–	–	–	0.02	0.02
*Bradyrhizobium* sp.	0.36	–	–	–	0.02	–
Chicken intestinal bacterium	0.16	–	–	0.04	–	7.63
*Clostridia bacterium*	0.06	0.06	0.01	0.03	0.13	0.10
*Corynebacterium auriscanis*	0.13	–	0.06	–	–	0.02
*Corynebacterium canis*	10.40	–	0.17	–	0.36	–
*Corynebacterium felinum*	1.74	–	0.04	–	0.04	–
*Corynebacterium* sp.	0.07	–	–	0.02	–	0.18
*Firmicutes* bacterium	0.02	0.03	0.01	0.02	0.11	0.03
*Haemophilus* sp.	–	0.04	0.09	0.02	0.13	–
*Klebsiella oxytoca*	0.36	–	–	–	0.04	–
*Klebsiella* sp.	1.34	0.03	0.04	–	–	–
*Kocuria carniphila*	2.85	–	0.06	–	0.04	–
*Kocuria* sp.	0.23	–	0.01	–	–	–
*Lactobacillus fermentum*	0.59	–	–	0.99	–	–
*Lactobacillus paraplantarum*	0.02	0.04	0.04	0.02	0.02	0.08
*Lactobacillus salivarius*	1.77	–	0.04	0.55	–	70.46
*Lactobacillus* sp.	0.17	–	0.01	0.21	–	0.01
*Lactococcus lactis*	0.58	–	–	–	–	–
*Macrococcus* sp.	0.32	–	–	–	–	–
*Pseudomonas* sp.	0.12	0.08	–	–	–	–
*Sphingobacterium* sp.	0.14	–	–	–	–	–
*Staphylococcus* sp.	0.17	–	–	–	–	0.01
*Stenotrophomonas maltophilia*	0.27	0.02	–	–	0.02	–
*Streptococcus alactolyticus*	0.23	95.55	92.35	92.34	92.47	–
*Streptococcus minor*	0.07	0.03	0.03	0.02	0.02	–
*Streptococcus pneumoniae*	0.05	0.14	0.34	0.11	0.41	0.08
*Streptococcus salivarius*	–	0.04	0.17	0.06	0.13	–
*Streptococcus* sp.	0.06	0.15	0.66	0.14	0.47	–
Uncultured bacteria	34.51	3.24	5.02	4.81	4.82	20.76
*Yokenella regensburgei*	1.60	0.02	0.01	–	–	–
Others	3.43	0.51	0.30	0.59	0.19	0.60

The values represent the percentage of the total reads belonging to the different species in each sample. Only those bacterial species that reached, at least, 0.1% of the total reads are displayed. M, gall bladder mucus layer; B, gall bladder biopsy.

We have also determined the inferred metagenomes from phylogenetically associated reference genomes in four bile samples. Predicted metagenomes at the three hierarchical KEGG pathway levels allowed us to infer the functional categories represented in the bile microbiota. Membrane transport, amino acid and carbohydrate metabolism accounted for more than one third of the hypothetical functions from the KEGG pathways at level 2 (Table S1).

### Proteomic analysis

**Figure 5:**
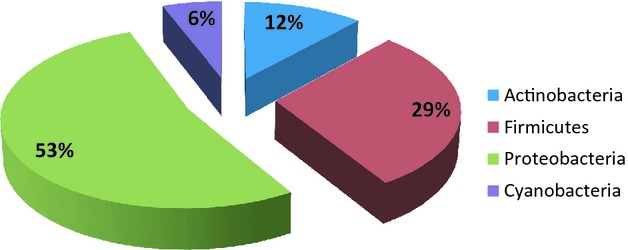
Bacterial phyla as deduced from the taxonomic analysis of biliary proteins.

A total of 214 proteins were identified in five bile samples. Of them, 129 were multiple-hit proteins and 85 were identified with one peptide in at least two injections (Table S2). Among all entries, 22 proteins were specifically associated to the Bacteria superkingdom (Table S3), 191 were from the *Sus scrofa* species, and 1 was found ambiguously associated to both taxonomic ranks. The subsequent classification of bacterial proteins according to the phylum of the source microorganisms revealed that most of them belonged to the *Proteobacteria* (53%). Other represented phyla included *Firmicutes* (29%), *Actinobacteria* (12%), and *Cyanobacteria* (6%) (Fig. 5). Unfortunately, any further analysis of the bacterial genera and species was hampered by the inadequate number of unique bacterial peptides, probably due to the interference of most abundant pig proteins.

## Discussion

In this study, the presence of live bacteria and bacterial DNA in bile and gall bladder samples from healthy sows was evidenced by culture-dependent and -independent techniques, respectively. In addition, the presence of bacterial proteins was also confirmed in bile samples. To the best of our knowledge, this is the first microbiome and proteomic study focused on bile and gall bladder of healthy hosts.

The combination of different techniques provided highly complementary views of the microbiota present in the analyzed samples. All the bacterial genera and most of the species isolated using culture techniques were later detected by pyrosequencing techniques although, obviously, the microbiome approach allowed the detection of a wider bacterial diversity. Globally, *S. alactolyticus* seemed to be the dominant species accounting for more than 90% of the reads in samples 3 and 4. This species was originally described among isolates obtained from the GIT of pigs (Farrow et al. 1984) and, since then, has been reported as a common member of the commensal gut microbiota in this host (Devriese et al. 1994; Højberg et al. 2005). A recent study on the microbial communities in the tonsils of healthy pigs has also shown strong similarities in the major components as well as distinct differences in minor components of the microbiome of this highly specialized site (Lowe et al. 2012). It is worth mentioning that the most abundant phyla detected using culture-dependent techniques (*Actinobacteria*, *Proteobacteria*, and *Firmicutes*) were coincident with the main phyla identified with the pyrosequencing analysis, and also with the most abundant bacterial proteins detected in the proteomic analysis, thus supporting the soundness and reliability of our results. Remarkably, members of the phylum *Bacteroidetes* were hardly detected in the bile samples analyzed. Therefore, our results suggest that *Bacteroidetes* are not adapted to a bile rich habitat, although it has been shown that they are among the most abundant phyla in the swine intestine (Lamendella et al. 2011; Looft et al. 2012; Wu et al. 2012).

Bile acids are among the major organic compounds of bile. They are amphipathic molecules with a strong antimicrobial activity (Sánchez et al. 2005; Kurdi et al. 2006) and, since about 5% of the total bile salts escapes active transport in the distal ileum and reaches the colon, they can exert a strong selective pressure on the gut microbiota (Margolles and Yokota 2011). It is becoming evident, indeed, that bile composition shapes the profile of our intestinal microbiota (Islam et al. 2011). Recent research suggests that dietary fat can alter bile composition, through conjugation of hepatic bile acids, and so favor the growth of proinflammatory gut microbes (Swann et al. 2011; Devkota et al. 2012). The results of this study confirm that bile-associated microorganisms are related to gut microbiota populations, although how such bile inhabitants exert an influence on the gut microbiota remains unknown. Inversely, bacteria normally present in the gut can invade the biliary tract by ascending from the duodenum or by the hematogenous route from the hepatic portal venous blood (Kook et al. 2010). Ecological niches in the human microbiome are not thought to be isolated environments, but rather a network of interrelated communities experiencing constant exchange (Costello et al. 2009). Therefore, it is very likely that gall bladder and gut bacterial communities are not an exception, and that they exert constant influences on each other.

The antimicrobial activity of bile salts may be one of the reasons explaining why bacterial diversity within the gall bladder-associated microbiome seems to be much lower than that observed in the gut of this species (Lamendella et al. 2011; Isaacson and Kim 2012). Therefore, the components of the autochthonous bile microbiota must have specific resistance mechanisms, including specific metabolic properties, to counteract the deleterious action of these toxic metabolites, which are extremely concentrated (100–200 mmol/L) in the gall bladder. Aside from specific bile-resistance mechanisms (including bile efflux systems and bile salt hydrolases), some bacteria are able to assimilate and metabolize some components of bile (Begley et al. 2005a, 2005b; Ridlon et al. 2006; Jones et al. 2008, 2012; Dowd et al. 2011; Park et al. 2013). Also, general stress response mechanisms, such as proton pumps, chaperones and proteases, are involved in bile detoxification (Begley et al. 2005a, 2005b; Sánchez et al. 2006). In our study, 7 of 22 bacterial proteins identified are different subunits of the ATP synthase (Table S3). This enzymatic complex is involved in bile tolerance of intestinal bacteria (Sánchez et al. 2006), and our data suggest that it could play a key role to allow survival of bile-adapted microbiota in the gall bladder.

Interestingly, the antibacterial effects of bile depend on the pH of the bile's environment. It has been previously reported that bile is not a hostile environment for bacterial growth when its pH value is similar to that naturally found in gall bladder (pH ˜7), supporting the growth of a wide spectrum of Gram-negative and Gram-positive species without the requirement of specific molecular detoxifying systems (Dowd et al. 2011). However, as bile from the gall bladder is released into the duodenum, it mixes with chyme from the stomach, thereby reducing the local pH to as low as pH 5.2. Under such circumstances, bile becomes toxic and *L. monocytogenes* require Sigma B-regulated protein systems (BSH, BilE, and Sigma B itself) to survive (Dowd et al. 2011).

The microbiological profiles of the samples obtained from the different animals show a high interindividual variability. All the sows were from the same farm and genetic lineage but from different litters. We know very little about the microbial inhabitants of bile, their metabolism and enzymatic activities, and its relationship with the diet, bile-related disorders, or any other factor that may play a role in its composition. The current knowledge is limited to a few species of cultivable bacteria that have been associated with gall bladder infections or gallstones formation (Hardy et al. 2004, 2006; Crawford et al. 2008, 2010; Gonzalez-Escobedo et al. 2011), but in few occasions in healthy hosts (Kook et al. 2010). Enterobacteria (*Salmonella*, *Klebsiella*, *E*. *coli*) and *L. monocytogenes* can replicate in the gall bladder and seem to be involved in the abovementioned pathogenic conditions; this is in contrast with the results of this study where streptococci, staphylococci, and related Gram-positive bacteria, including lactobacilli, could be isolated or detected by pyrosequencing. This fact rises the hypothesis that the gall bladder contains a site-specific microbiota that can be disturbed by factors that, at present, are unknown, and that such dysbiosis process may be linked to gall bladder pathologies. This is a relevant health issue since more than one million of cholecystectomies (removal of the gall bladder) are performed per year only in Europe, with associated direct costs of more than one billion € (Sauerland 2008). Therefore, the study of the gall bladder-associated microbiota and its implication in gall bladder and gut health deserves further research.

## Supporting Information

Table S1 KEGG pathways in level 2 in bile samples (B2–5).

Table S2 Total proteins identified in bile samples (B1–5).

Table S3 Bacterial proteins identified in bile samples (B1–5).
